# Outcome of giant pituitary tumors requiring surgery

**DOI:** 10.3389/fendo.2022.975560

**Published:** 2022-08-29

**Authors:** Stephan Gaillard, Sosthène Adeniran, Chiara Villa, Anne Jouinot, Marie-Laure Raffin-Sanson, Loic Feuvret, Pierre Verrelle, Fidéline Bonnet, Anthony Dohan, Jérôme Bertherat, Guillaume Assié, Bertrand Baussart

**Affiliations:** ^1^ Department of Neurosurgery, Assistance Publique-Hôpitaux de Paris, La Pitié-Salpêtrière University Hospital, Paris, France; ^2^ Department of Neurosurgery, Centre Hospitalier Universitaire Yalgado Ouédraogo University Hospital, Ouagadougou, Burkina Faso; ^3^ Université Paris Cité, Institut Cochin, CNRS, INSERM, Paris, France; ^4^ Department of Pathological Cytology and Anatomy, Assistance Publique-Hôpitaux de Paris, La Pitié-Salpêtrière University Hospital, Paris, France; ^5^ Department of Endocrinology, Centre Hospitalier de Liège, Université de Liège, Domaine Universitaire du Sart Tilman, Liège, Belgium; ^6^ Institut Curie, INSERM, MINES ParisTech, PSL-Research University, CBIO-Centre for Computational Biology, Paris, France; ^7^ Department of Endocrinology, Assistance Publique-Hôpitaux de Paris, Hôpital Ambroise Paré, Boulogne Billancourt, France; ^8^ Université de Versailles Saint-Quentin-en-Yvelines UFR des Sciences de la Santé Simone Veil, Montigny-le-Bretonneux, France; ^9^ Radiation Oncology Department, Assistance Publique-Hôpitaux de Paris, La Pitié-Salpétrière University Hospital, Paris, France; ^10^ Radiation Oncology Department, Institut Curie, Paris, France; ^11^ Assistance Publique Hôpitaux de Paris, Hôpital Cochin, Hormonal Biology Laboratory, Paris, France; ^12^ Department of Radiology, Assistance Publique-Hôpitaux de Paris, Hôpital Cochin, Paris, France; ^13^ Department of Endocrinology, Assistance Publique-Hôpitaux de Paris, Hôpital Cochin, Center for Rare Adrenal Diseases, Paris, France

**Keywords:** giant pituitary tumor, adenoma, surgery, complication, radiotherapy, aggressiveness

## Abstract

**Objective:**

The management of giant pituitary tumors is complex, with few publications and recommendations. Consequently, patient’s care mainly relies on clinical experience. We report here a first large series of patients with giant pituitary tumors managed by a multidisciplinary expert team, focusing on treatments and outcome.

**Methods:**

A retrospective cohort study was conducted. Giant pituitary tumors were defined by a main diameter > 40mm. Macroprolactinomas sensitive to dopamine agonists were excluded. All patients were operated by a single neurosurgical team. After surgery, multimodal management was proposed, including hormone replacement, radiotherapy and anti-tumor medical therapies. Outcome was modeled using Kaplan-Meyer representation. A logistic regression model was built to identify the risk factors associated with surgical complications.

**Results:**

63 consecutive patients presented a giant adenoma, most often with visual defects. Patients were operated once, twice or three times in 59%, 40% and 1% of cases respectively, mainly through endoscopic endonasal approach. Giant adenomas included gonadotroph, corticotroph, somatotroph, lactotroph and mixed GH-PRL subtypes in 67%, 14%, 11%, 6% and 2% of patients respectively. Vision improved in 89% of patients with prior visual defects. Severe surgical complications occurred in 11% of patients, mainly for tumors > 50 mm requiring microscopic transcranial approach. Additional radiotherapy was needed for 29% of patients, 3 to 56 months after first surgery. For 6% of patients, Temozolomide treatment was required, 19 to 66 months after first surgery.

**Conclusions:**

Giant pituitary tumors require multimodal management, with a central role of surgery. Most often, tumor control can be achieved by expert multidisciplinary teams.

## Introduction

Pituitary adenomas account for 15% of all intracranial tumors (1). Giant pituitary adenomas are defined as adenomas with a main diameter > 40 mm (2–5). Giant pituitary adenomas represent 8% of all pituitary adenomas and occur predominantly in males (5, 6). Non-functioning giant macroadenomas, including gonadotropin-secreting, silent corticotroph and null-cell adenomas, account for approximately 70% of all encountered subtypes, followed by prolactin-secreting (20%), GH and mixed GH-PRL (10%), while TSH secreting adenomas are exceptional (2, 6–8).

Giant adenomas are usually revealed by visual deterioration, endocrine dysfunction or cognitive disorders (9). Except for giant prolactinomas and rare giant somatotroph adenomas with a significant response to somatostatin analogues, surgery is usually indicated, with the main objective of visual pathways decompression and maximum volume reduction (8, 10–13). For giant prolactinomas, the first-line treatment is dopamine agonists, with an excellent efficiency in most cases (7, 14–16). In rare cases of giant prolactinomas with resistance to dopamine agonists treatment, pituitary surgery can be positioned as a second-line therapy (7, 14, 17).

Pituitary surgery of giant adenomas is challenging, and require high expertise (3, 18–21). Surgery of giant adenomas is associated with a higher rate of postoperative complications, compared to non-giant pituitary macroadenomas (4, 8, 11, 22–25). This increased risk is related to more complex dissection techniques, the potential need of multiple surgeries, and the potential use of extended endoscopic trans-tuberculum and transcranial approaches in addition to trans-sellar approaches (10, 18, 19, 26).

Surgery of giant adenomas is almost never complete. Indeed, invasion of cavernous sinus, lateral or posterior extensions towards temporal lobe and brainstem are common. Additional treatments are often needed, including radiotherapy and medical therapies (4, 6, 27). In some giant adenomas, a pejorative course can be observed, with rapid tumor growth despite these additional therapies. On the basis of invasiveness and tumor proliferation, clinicopathological classifications have been developed to predict such aggressive behavior (28–30). However, the definition of aggressiveness remains to be established. In this context, the term PitNETs has recently been proposed to recall pituitary adenomas (31, 32) but its widespread use is still debated (33).

The global therapeutic strategy is discussed upfront surgery in a multidisciplinary expert setting. After surgery, a long-term management is required, including hormone replacements, anti-tumor treatments, and surveillance for any tumor progression.

Currently, few large series with > 50 patients treated by endoscopic approach are available, with limited information on outcome (3, 4, 8, 10, 11, 25, 34, 35). The aim of the present study is to report a large consecutive series of patients with a giant pituitary adenoma, focusing on the initial presentation, the surgical management, the pathology findings, the surgical complications, the post-operative multimodal strategy and the long-term outcome.

## Materials and methods

### Patients

In this observational cohort, 63 consecutive patients with a giant macroadenoma operated on between January 2010 and October 2020 were included.

The diagnosis of a giant macroadenoma was based on: (i) a largest diameter > 40 mm on MRI; (ii) a histological confirmation on pathological analysis.

All patients with a prior adenoma surgery or a prior history of radiotherapy were excluded from this series.

An ophthalmic examination with an acuity and visual field analysis was performed by a senior referent ophthalmologist before, 3 months after surgery, and during follow-up.

For all patients, pituitary function was assessed by a referent endocrinologist and included the hormonal assessment of each pituitary axis.

The surgical decision was taken by a multidisciplinary team gathering endocrinologists, neurosurgeons, and radiologists. For macroprolactinomas, surgery was only indicated in cases of resistance to prior treatment by dopamine agonists. In accordance with the French legislation, patient consent was not needed for this retrospective noninterventional study evaluating a routine care. A local ethical agreement was obtained by the Ethical Review Committee for publications of the Cochin University Hospital (CLEP, N°: AAA-2022-08022).

### Pituitary MRI evaluation

**Figure 1:**
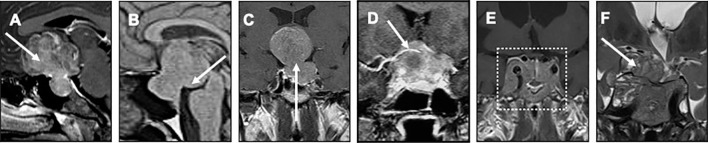
Different patterns of giant tumors extension Preoperative pituitary MRI are presented, including gadolinium-enhanced T1 weighted sagittal **(A, B)**, coronal **(C–E)** and T2 weighted coronal **(F)** images. **(A)** subfrontal extension (white arrow); **(B)** posterior fossa extension (white arrow); **(C)** suprasellar extension with a “narrow neck” aspect between the intrasellar and the suprasellar tumor components; **(D)** encasement of the right anterior cerebral artery (white arrow); **(E)** massive invasive sphenoid tumor bridging the two cavernous sinuses, defined as “sphenoid arch” aspect (white dotted rectangle); **(F)** tumor extension through the roof-top of the right cavernous sinus (white arrow).

All patients underwent a preoperative dedicated pituitary MRI including T1 and T2-weighted spin echo sequences (axial, sagittal and coronal) before and after intravenous gadolinium chelate injection, read by a senior radiologist. Specific radiological patterns were noted, as provided in **Figure 1**.

For all tumors, the largest diameter was reported. Cavernous sinus invasion was documented on both sides on the basis of Knops grading (36). Invasion was further detailed, focusing on potential intradural cisternal extension through the roof-top of cavernous sinus (**Figure 1F**), and potential massive sphenoid invasion bridging the two cavernous sinuses (referred to as “sphenoid arch”, **Figure 1E**). When occurring, encasement of a cerebral artery (defined as a tumor component totally surrounding one of the arteries of the circle of Willis, **Figure 1D**) and hypothalamic hypersignal on T2-weighted sequences were reported.

Tumor extension was assessed in all directions: inferior infrasellar, superior suprasellar (either null, tumor bulging into the chiasmatic cistern or in contact with the optic chiasma but with no compression, or compression of the optic chiasma), anterior subfrontal (**Figure 1A**), posterior towards the brainstem **(** **Figure 1B**), and lateral towards the temporal lobe. Situations of large suprasellar extension separated from a small intrasellar component by a narrow neck were noted (referred to as “narrow neck**”,** **Figure 1C**). Hydrocephalus was reported whenever occurring.

Three months after surgery, resection was evaluated by MRI. Resection was considered either as complete if no residue was observed; as subtotal if the percentage of residual tumor volume was ≤ 20% of the preoperative tumor volume or partial if the percentage of residual tumor volume was > 20%.

During follow-up, MRI controls were repeated every 6 to 12 months, to track down tumor progression.

### Surgery

All patients were operated on by the same two expert senior neurosurgeons (S.G, B.B). The main surgical objectives were maximum tumor volume debulking, decompression of the visual pathways, and the optimization of target volume for potential secondary radiotherapy.

**Figure 2:**
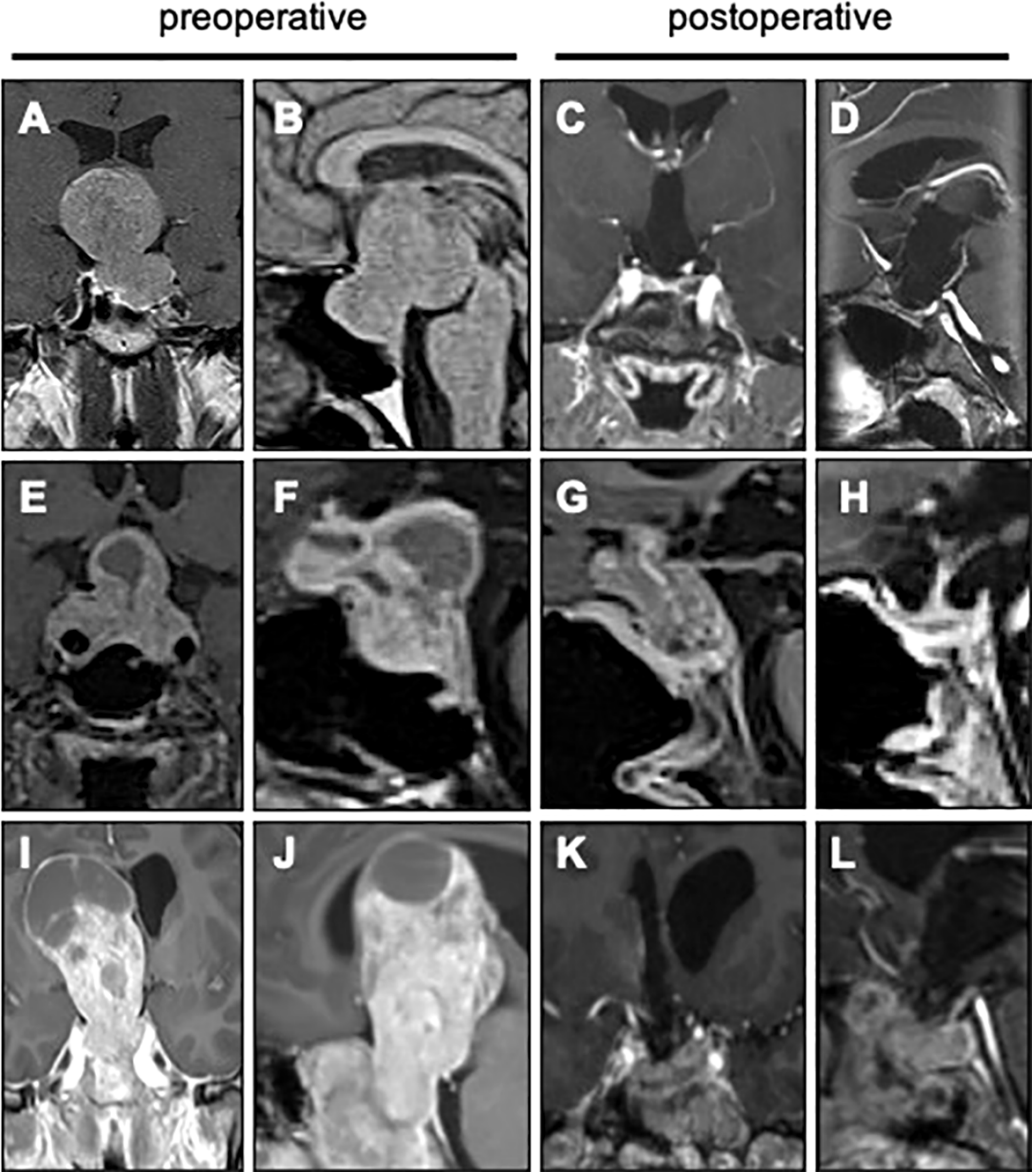
Surgical resection of giant pituitary tumors Pituitary MRI from three patients are presented, before and after surgery (gadolinium-enhanced T1 weighted coronal and sagittal images). **(A-D)** Giant pituitary tumor with a “narrow neck” pattern, resected with an extended endoscopic endonasal approach. **(E-H)** Giant pituitary tumor with transdiaphragmatic subfrontal extension, resected in two steps with combined endoscopic endonasal and microscopic transcranial approaches. **(I-L)** Giant pituitary tumor with a huge suprasellar expansion extending beyond the Monro foramen, resected with a transfrontal transcranial approach.

Whenever possible, the first line approach was the mononostril endoscopic endonasal transsphenoidal approach, as performed for common pituitary tumors (37). In a few cases, the resection of the suprasellar extension could be achieved by an extracapsular dissection with a binostril trans-tuberculum expanded endonasal approach (**Figures 2A–D**), followed by a multilayer skull base reconstruction with nasoseptal mucosal pedicled flaps, as previously reported (5, 38). In patients with upper remnant, a second transsphenoidal surgery was proposed each time the remnant spontaneously dropped into the sellar area after 3 months.

Whenever a large debulking was unachievable or insufficient by transsphenoidal approach, a microscopic transcranial approach could be decided (**Figures 2E–H**), by pterional approach in all patients except for one case of transcortical transfrontal approach (**Figures I–L**). This situation corresponds mainly to subfrontal or lateral extensions, extension through the roof-top of cavernous sinus, encasement of a cerebral artery, or some giant suprasellar extensions with “narrow neck” aspect, as provided in **Figure 1C**.

Hydrocephalus was treated specifically. For acute hydrocephalus, external ventricular drainage or ventriculoperitoneal shunt were performed. For delayed hydrocephalus, a ventriculoperitoneal shunt was performed.

Standard peri- and post-operative hydrocortisone supplementation were given to all patients. All patients received thromboprophylaxis. In the absence of postoperative complications, patients were discharged on the 5th day post-surgery with the approval of the endocrinologist. In case of postoperative complications, the hospitalization could be longer, depending on the outcome.

Immediate surgical complications were noted during the stay at the neurosurgical department: death, vascular injury, ischemia, hematoma, visual deterioration, neurological deficit, postoperative CSF leakage, meningitis, sinonasal disorders. Postoperative persistent neurological deficits (related to postoperative ischemia, traumatic surgical dissection or hematoma), hematomas requiring a second surgery, meningitis and surgical site infections were defined as severe complications.

### Pathology

All tumor samplings were histopathologically studied by immunohistological essay. Each specimen was stained for hormonal markers (PRL, GH, TSH, ACTH, FSH, LH).

Proliferation index was measured by Ki67 immunostaining and mitotic count. Nuclear p53 immunopositivity was also evaluated. For each patient, tumor grade was defined following the Lyon’s clinicopathological classification (29, 30), with grades 1a, 2a, 2b corresponding to non-invasive and non-proliferative tumors, invasive non-proliferative, and invasive-proliferative tumors respectively.

### Patients’ management beyond surgery

Before and after initial surgery, a multidisciplinary management was proposed, with expert endocrinologists, neurosurgeons, radiologists, radiotherapists and oncologists.

After surgery, medical treatments were proposed either in case of persistent hypersecretion, including somatostatin analogues in somatotroph tumors, dopamine agonists in prolactinomas, and pasireotide and/or cabergoline in corticotroph tumors.

Radiotherapy was generally proposed for patients with remnants growing after surgery, either directly, or after trying a medical treatment in case of slow growth (with somatostatin analogues for somatotroph adenomas, with pasireotide and/or cabergoline for corticotroph tumors). For patients with potential aggressive pathological features, an adjuvant radiotherapy was proposed shortly after surgery. Radiotherapy, including conformational photon radiotherapy and proton radiotherapy, targeted a large tumor volume, encompassing the whole tumor area.

Chemotherapy was proposed in general in case of rapid growth in spite of prior radiotherapy, following the clinical practice guidelines for the management of aggressive pituitary tumors. Temozolomide was the first line chemotherapy (29). In patients with prolactinomas, temozolomide was combined with cabergoline. In patients with corticotroph adenomas, temozolomide was combined with pasireotide.

### Statistical analyses

The following qualitative variables were collected for each patient: sex, vision deficit, cavernous sinus symptoms, hypersecretion-related symptoms, biological hypersecretion, hormonal deficit, hydrocephalus, MRI invasion, suprasellar extension, suprasellar extension with “narrow neck” aspect, infrasellar extension, posterior extension, subfrontal extension, extension through the rooftop of a cavernous sinus, encasement of cerebral artery, “sphenoid arch” aspect, MRI T2 hypothalamic hyperintensity, number of surgeries, type of surgery, tumor consistency, vision improvement, quality of resection, visual pathways decompression, pathological histotype, tumor grade, new postoperative anterior insufficiency, diabetes insipidus, postoperative cerebrospinal fluid leakage, postoperative stroke, postoperative hematoma, postoperative meningitis, visual deterioration, cognitive deterioration, motor deterioration. The following qualitative variables were collected for each patient: age, maximum diameter, KI67, mitotic count, p53, follow-up after first surgery, follow-up after radiotherapy.

Statistical analysis was performed using R statistical software (version 3.6.3, survival and survcomp packages). Descriptive statistics used median (range) for quantitative variables and raw numbers (%) for categorical variables. Comparisons between groups were performed using Fisher’s exact test for categorical variables.

Cumulative curve of event and disease-free survival were achieved using Kaplan–Meier estimates. Logistic regression was used to test the association of selected features (age, sex, visual acuity, MRI characteristics, number and type of surgeries) with surgical complications. P-values (P) level of significance was set at P< 0.05.

## Results

### Patient characteristics

The characteristics of 63 patients operated on for a giant pituitary macroadenoma are provided in **Tables 1**, **2**. Patients were mostly men (F/M ratio: 0.58) with a median age of 54.4 (range: 19.2 – 79.3). Visual deficiency was observed in 56/63 (89%) of patients with a median lowest visual acuity of 2/10 (range: 0 – 10), while cavernous sinus and cognitive symptoms were noted in 3/63 (5%) and 4/63 (6%) of patients respectively. Hormone excess was observed in 14/63 (22%) of patients, mostly with somatotroph hypersecretion (50%), but also lactotroph (29%), corticotroph (14%) and mixed GH/PRL (7%). Pituitary deficiency was observed in 42/63 (67%) of patients.

**Table 1:** Preoperative characteristics of 63 patients treated for a giant pituitary adenoma.

	All patients N = 63
**Age**, years	54.4 (19.2-79.3)
**Sex**	
Female Male	23 (37%) 40 (63%)
**Tumoral Symptoms**	**59 (94%)**
Visual symptoms	56 (89%)
Cavernous sinus symptoms	3 (5%)
Cognitive symptoms	4 (6%)
**Pituitary Deficiency**	**42 (67%)**
One axis	13 (21%)
Two axes	10 (16%)
Panhypopituitarism	19 (30%)
**Hormonoe Excess**	**14 (22%)**
Corticotroph hypersecretion	2 (3%)
Somatotroph hypersecretion	7 (11%)
Mixed GH-PRL hypersecretion	1 (2%)
Lactotroph hypersecretion	4 (6%)
**MRI aspect**	
Maximum diameter	46 (41-72)
Hydrocephalus	4 (6%)
Invasiveness	61 (97%)
Suprasellar extension	
No visual pathways compression	7 (11%)
Visual pathwaus compression	56 (89%)
Infrasellar extension	34 (54%)
Posterior extension	7 (11%)
Encasement of a cerebral artery	22 (35%)
Sphenoid arch aspect	4 (6%)
Extension through the roof top of a cavernous sinus	12 (19%)
T2 hypothalamic hyperintensity	5 (8%)
Suprasellar extension with “narrow neck” aspect	12 (19%)

Quantitative variables are expressed in median (range); qualitative variables are expressed in absolute numbers (proportion). In the bold value of the heading, the data are expressed in absolute numbers (N).

**Table 2:** Peri-operative characteristics of 63 patients treated for a giant pituitary adenoma.

	N	Number of Patients
**Tumor consistency**	63	
Soft		38 (60%)
Fibrous		25 (40%)
**Number of surgeries**	63	
1		37 (59%)
≥2		26 (41%)
**Type of surgery**	63	
Endonasal only		46 (73%)
Transcranial +/- endonasal		17 (27%)
**Visual Improvement** No Yes (at least one eye)	56	6 (11%) 50 (89%)
**Quality of resection**	63	
Complete		2 (3%)
Subtotal		36 (57%)
Partial		25 (40%)
**Visual pathways decompression**	59	50 (85%)
**Subtype on pathology**	63	
Gonadotroph		42 (67%)
Corticotroph		9 (14%)
Somatotroph		7 (11%)
Lactotroph		4 (6%)
Mixed GH-PRL		1 (2%)
**Proliferative markers**		
KI 67	62	3 (1-30)
Mitoses	60	1 (0-40)
P53	12	0 (0-15)
**Lyon’s clinicopathological classification** 1a 2a 2b	47	1 (2%) 37 (79%) 9 (19%)

Quantitative variables are expressed in median (range); qualitative variables are expressed in absolute numbers (proportion).

On preoperative MRI, the median main diameter was 46 mm (range: 41 – 72). Except for 2 patients, all giant adenomas invaded obviously surrounding structures (97%). Cavernous sinus was invaded in 59/63 (94%) patients. All patients had suprasellar extensions, with compression of the optic chiasm in 56/63 (89%) of cases. Specific tumor patterns including encasement of cerebral artery, massive sphenoid invasion bridging the two cavernous sinuses, intradural extension through the roof-top of a cavernous sinus, “narrow neck” aspect and T2 hypothalamic hyperintensity were observed in 22/63 (35%), 4/63 (6%), 12/63 (19%), 12/63 (19%) and 5/63 (8%) of patients respectively.

### Initial surgery

All patients were operated. Four patients were operated in the context of acute hydrocephalus. An external ventricular drainage was performed in 2/4 (50%) patients, with no need for any additional ventriculoperitoneal shunt afterwards.

First surgery was performed by endoscopic endonasal approach or microscopic transcranial approach in 57/63 (90%) and 6/63 (10%) patients respectively.

During surgery, tumor consistency was soft in 38/63 (60%) of cases. In 25/63 (40%) of cases, tumor consistency was fibrous, increasing the complexity of surgical resection.

On pathological analysis, gonadotroph adenomas were found in the majority of cases (n=42/63, 67%). Corticotroph, somatotroph, lactotroph secreting, and mixed GH-PRL secreting adenomas were found in 9/63 (14%), 7/63 (11%), 4/63 (6%) and 1/63 (2%) of patients respectively. Considering the 2 patients with biological corticotroph hypersecretion, silent corticotroph adenoma were therefore observed in 7/63 (11%) of patients.

### Complications related to surgery

Surgical complications occurred in 13/63 (21%) of patients (**Table 3**). Visual deterioration was noted in 3/63 (5%) patients. Motor and cognitive deterioration was observed in 5/63 (8%) and 3/63 (5%) patients, but the neurological examination recovered in 3/5 (60%) and 1/3 (33%) patients respectively. No carotid or anterior choroidal artery injury was observed, but stroke due to perforating artery injury occurred in 4/63 (6%) patients. Overall, severe surgical complications were observed in 7/63 (11%) patients, including meningitis, hematoma requiring second surgery and stroke-related persistent deficiencies in 3/63 (5%), 2/63 (3%) and 3/63 (5%) cases respectively. Severe complications were observed in 5/22 (23%) patients with a tumor larger than > 50 mm and 5/17 (29%) patients treated by microscopic transcranial approach. For patients treated with endoscopic endonasal approach, postoperative CSF leakage was diagnosed in 4/60 (7%) of patients, requiring a second surgery in all cases.

**Table 3:** Complications and endocrine consequences of pituitary surgery Data are expressed as N (%).

	All PatientsN = 63	Endoscopic Endonasal ApproachN=60	Microscopic Transcranial ApproachN=17
**Surgical Complication**			
Vascular injury Carotid artery injury Anterior choroidal artery injury Perforating artery	4 (6%) 0 0 4 (6%)	0	4 (24%) 0 0 4 (24%)
Hematoma Asymptomatic Symptomatic requiring surgery	6 (9%) 4 (6%) 2 (3%)	2 (4%) 1 (2%) 1 (2%)	4 (24%) 3 (18%) 1 (6%)
Visual deterioration Monocular Binocular	3 (5%)	0	3 (17%) 1 (6%) 2 (11%)
Motor deterioration Transient Persistent	5 (8%) 3 (5%) 2 (3%)	0	5 (29%) 3 (17%) 2 (12%)
Cognitive deterioration Transient Persistent	3 (5%) 1 (2%) 2 (3%)	0	3 (17%) 1 (6%) 2 (11%)
Cranial nerve palsy	3 (5%)	0	3 (18%)
Postoperative CSF leakage requiring plasty	4 (6%)	4 (7%)	0
Meningitis/Infection	3 (5%)	1 (2%)	2 (11%)
Epistaxis, rhinitis, sinusitis	1 (2%)	1 (2%)	0
**Total complications** Severe complications*	**13 (21%)** **7 (11%)**	**6 (10%)** **2 (3%)**	**7 (41%)** **5 (29%)**
**Endocrine consequences of pituitary surgery**			
Anterior pituitary insufficiency One axis Two axes Panhypopituitarism	8 (13%) 6 (9%) 1 (2%) 1 (2%)		
Diabetes insipidus Transient Persistent	15 (24%) 8 (13%)		

Qualitative variables are expressed in absolute numbers (proportion).

*Severe complications included postoperative persistent neurological deficits (related to postoperative ischemia, traumatic surgical dissection or hematoma), hematomas requiring a second surgery, meningitis and surgical site infections.

In the bold values, the data are expressed in absolute numbers (N).

Microscopic transcranial approach (OR=40.3 [7.1-228], P<0.001), larger main diameter (OR=1.2 [1.1-1.3], P<0.01), macroadenomas with encasement of cerebral artery (OR=4.1 [1.1-14.7], P<0.05), suprasellar extension with “narrow neck” aspect (OR=6.3 [1.6-25.1], P<0.01), hydrocephalus (OR=14.7 [1.4-156], P<0.05), number of surgeries ≥ 2 (OR=4.4 [1.2-16.3], P<0.05) were significant predictive factors of complications after surgery (**Table 4**).

**Table 4:** Logistic regression model predicting surgical complications.

Variables	Odds Ratio	95 CI	P-value
**Age/**year increase	1.01	0.97-1.05	0.663
**Sex (**Male vs Female)	2.22	0.54-9.09	0.267
**Lowest visual acuity**	1.03	0.86-1.23	0.763
**MRI**			
Main diameter (/mm increase)	1.18	1.05-1.31	0.00344*
Encasement of a cerebral artery	4.11	1.15-14.75	0.0299*
Suprasellar extension with “narrow neck” aspect	6.29	1.57-25.1	0.00925*
Hydrocephalus	14.7	1.38-156.19	0.0258*
**Number of surgeries** (≥ 2 vs 1)	4.37	1.17-16.27	0.028*
**Type of surgery** Microscopic transcranial vs Endoscopic endonasal only	40.33	7.14-227.78	2.84e-05*

*P < 0.05.

New anterior pituitary deficits were observed in 8/63 (13%) of patients, mostly affecting one pituitary endocrine axis. Diabetes insipidus occurred in 15/63 (24%) of patients and persisted in 8/63 (13%) of patients.

### Short term tumor volume control after surgery

Fifty-nine of 63 patients were operated in the context of visual defect. After surgery, radiological decompression of the visual pathways was obtained in 50/59 (85%) of patients, with vision improvement reported for 50/56 (89%) of patients.

One single surgery was sufficient to reach maximum tumor volume reduction in 37/63 (59%) of patients, while additional surgeries were needed for others, with two surgeries in 25/63 (40%), and three surgeries in 1/63 (1%). These 26 additional surgeries were performed by endoscopic endonasal approach or microscopic transcranial approach in 14/63 (22%) and 12/63 (19%) of patients respectively. After surgery, resection was complete in 2/63 (3%), subtotal in 36/63 (57%) and partial in 25 (40%) of patients. Complete resection was obtained for the two patients with non-invasive adenomas.

### Long term tumor volume control after surgery

**Figure 3:**
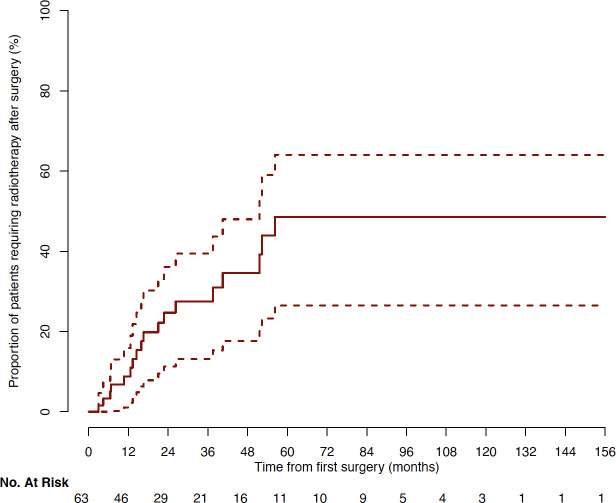
Cumulative incidence of patients requiring radiotherapy after surgery (Kaplan-Meyer representation).

**Figure 4:**
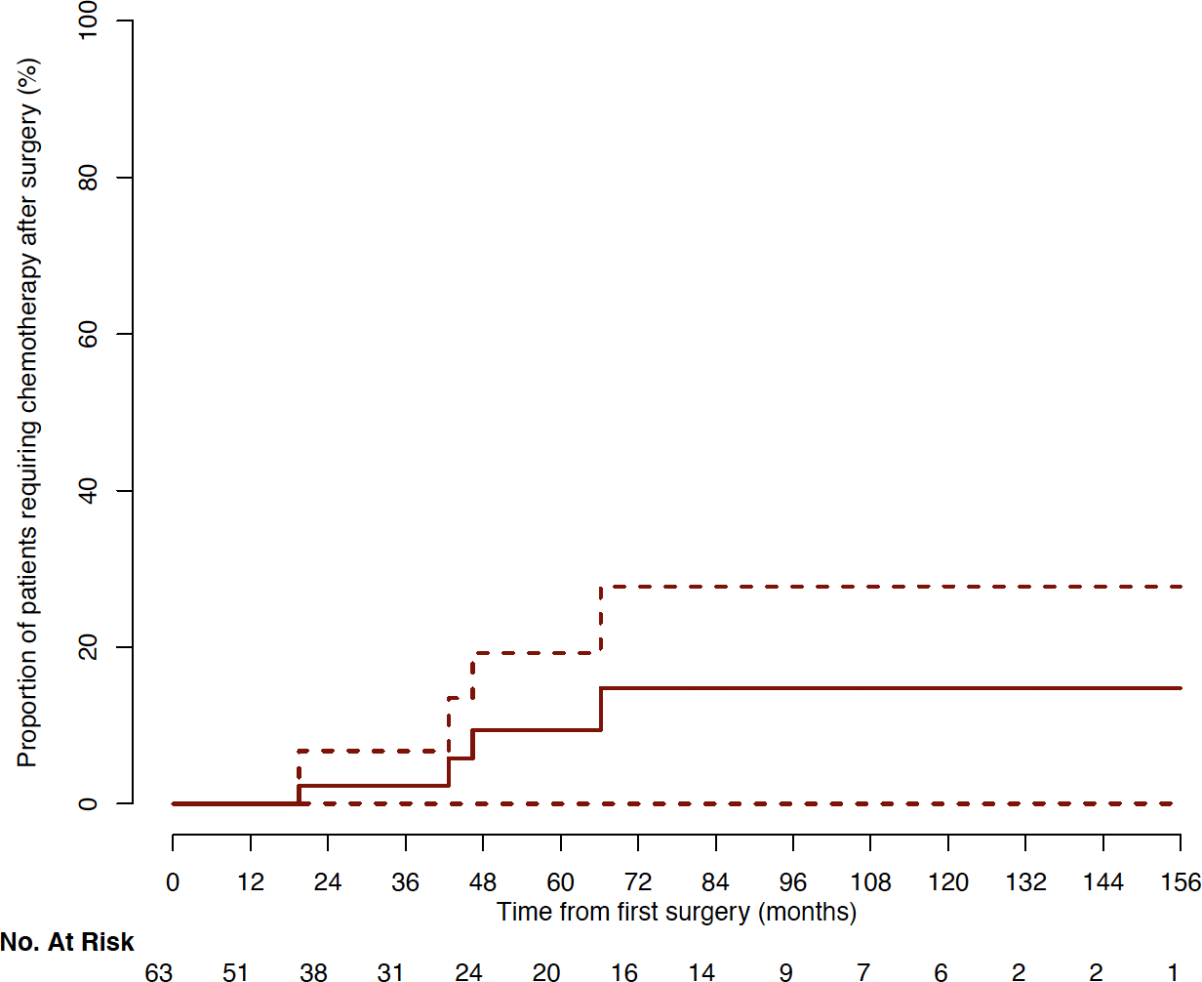
Cumulative incidence of patients requiring chemotherapy after surgery (Kaplan–Meyer representation).

Median follow-up was 32.8 months after initial surgery (range: 3.1 to 157.2). At last follow-up, pituitary surgery was sufficient to obtain tumor volume control in 45/63 (71%) of patients. For 18/63 (29%) of patients, radiotherapy was needed. The median time between surgery and radiotherapy was 16.2 months (range: 3 to 56.3, **Figure 3**). No radiotherapy was delivered beyond 56 months after surgery. At last follow-up, surgery alone or combined with radiotherapy, controlled disease progression in 59/63 (94%) of cases. The 4/63 (6%) remaining tumors were considered as aggressive tumors and required additional chemotherapy with Temozolomide with a median time of 44.6 months (range: 19.5 to 66.2) after first surgery (**Figure 4**), and 23.1 months (range: 5 – 42) after radiotherapy (**Supplementary Figure 1**).

### Giant tumors with pejorative evolution

Tumor progression occurred in four patients after radiotherapy, with a median time of 44.6 (range: 19.5 to 66.2) after first surgery. These four patients were treated with chemotherapy using Temozolomide. In comparison with the 59 patients controlled with surgery and radiotherapy, “sphenoid arch” invasion aspect (75 vs 2%, P<0.001), intradural extension through the cavernous sinus roof-top (100 vs 14%, P<0.001), high Ki67 rate (17.5 vs 3, P<0.01), 2B grades (75 vs 14%, P<0.05), were more common **(** **Tables 1**, **2** **).** Reversely, radical resection (0 vs 64%, P<0.05) and gonadotroph subtypes (29 vs 100%, P<0.01) were less common. No craniospinal or distant metastasis were observed.

Specific death due to uncontrolled tumor disease, relapsing despite chemotherapy, occurred in 2 out of the 4 patients.

## Discussion

This paper focuses on a large series of giant pituitary tumors requiring surgery. For giant dopamine agonists resistant prolactinomas and all other giant tumors, surgery is the standard of care as a first line treatment. We show here that surgery is able to control tumor volume in most patients, as supported by prior studies (5, 6, 8, 11, 23). In this study, a radical resection, including complete or subtotal resections, was obtained in 60% of patients, which may appear as a modest result compared to the higher rate observed for patients with smaller pituitary adenomas (20, 22, 39–42), but comparable to previous series (3, 8, 10, 11, 23, 34, 43–45). However, in the particular context of large invasions on surrounding structures and extension, the objective is rather the largest possible debulking than complete resection, due to the uttermost importance of limiting morbidity and complications. Despite the substantial rate of partial resection (40%), optic nerves and optic chiasm were decompressed in most patients (85%) on postoperative MRI, with an improvement of preoperative visual deficits in 89%. Taking into account the complexity of the surgical management, our study confirms that surgery should be considered as an efficient and safe first-line treatment for most giant macroadenoma patients in centers of expertise.

In the present study, 40% of giant pituitary adenomas were fibrous, contrasting with the 10% prevalence in non-giant tumors (46–48). Of note, as for all pituitary adenomas, the tumor consistency cannot be anticipated reliably by preoperative MRI data (47, 49). Fibrous consistency may increase the difficult of surgical dissection and may therefore affect the quality of resection. This may explain why the 10% rate of overall complications after endoscopic endonasal approach for giant pituitary adenomas resection is higher than the 3.5% rate we previously reported for non-giant pituitary adenomas (22). Nevertheless, this 10% rate compares favorably with the results described in previous studies (4, 8, 10, 11, 23, 34, 43, 45).

In this series, 59% of patients required more than one single surgery. Based on our significant experience of over 3000 pituitary adenomas treated by endoscopy, we are confident that debulking of most giant adenomas can be performed using a mononostril endonasal approach. However, a binostril approach obviously offers more working space. Thus, we recommend using the binostril approach early in the learning curve of pituitary endoscopy. For patients with huge but median suprasellar extensions, an expanded endoscopic endonasal approach can be decided, leading to a radical tumor resection in a one-step surgery, as previously published (5, 18). When suprasellar extension is more lateral than the carotid artery location, an additional microscopic transcranial approach is required to properly visualize and remove the lateral component. Moreover, resection by endoscopic endonasal dissection is limited for suprasellar trans-diaphragmatic extension with encasement of the carotid artery or anterior communicating complex, and the risk of complication is high. Therefore, in such cases, an additional transcranial approach is needed for an optimal optic nerve and chiasm decompression. In case of microscopic transcranial surgery, the quality of dissection is affected by the retrochiasmatic development of pituitary tumors. During transcranial approach, the neurosurgeon has to alternate different steps of tumor resection in narrow working windows while preserving all perforating arteries. Some perforating arteries (43, 50) could also be encased by the tumor, which explains the difficulty to preserve all arterial feeders during surgical dissection and the increased risk of postoperative ischemia (3, 43, 50–52). Finally, some trans-diaphragmatic extensions are not well encapsulated and small perforating arteries may run along the pseudo-capsule (24, 50, 53, 54). In such cases, an extracapsular dissection may damage theses functional arterial feeders and we recommend to perform an intratumoral debulking only. Unfortunately, in these cases of pure intracapsular debulking, the hemostasis may be more complex to obtain with an increased risk of postoperative symptomatic and asymptomatic hematoma. Despite these precautions, the rate of severe complications - including postoperative persistent neurological deficits, hematomas requiring a second surgery, meningitis and surgical site infections - after transcranial approach remains significant (29%), as supported by prior studies (8, 52, 55). For this reason, we can say that the use of transcranial approaches should be kept to a minimum and that alternative medical treatments should be preferred if possible.

Our study identified several preoperative MRI predictive factors of complications, including larger main diameter, cerebral artery encasement, suprasellar extension with a “narrow neck” aspect, or hydrocephalus. Some of these predictive factors have been reported by most prior studies (9, 11, 52). In clinical practice, the observation of pejorative preoperative features should lead to patients being informed about the increased risk of complications. In particular, considering the continuous risk increase with tumor diameter increase (OR 1.18 per mm), surgery should be decided fairly soon after the diagnosis of giant pituitary adenomas is made, and simple surveillance should be avoided.

In this study, 29% of patients were not controlled by surgery, and required radiotherapy. In contrast with prior studies (4, 10, 11), no radiotherapy was needed after surgery for 71% of patients. Here, radiotherapy was limited to patients with highly proliferative lesions, persistent remnants threatening the visual pathways, and growing remnants. The time between surgery and radiotherapy was highly variable. These indications were taken during multidisciplinary meetings, in accordance with current recommendations (2, 7, 18, 56, 57). Of note, when no radiotherapy was required beyond 5 years of follow-up, no further tumor progression was observed afterwards, and thus could be considered as long-term controlled by surgery.

In this cohort, 6% of patients displayed an aggressive course, a higher rate compared to the 0.5% prevalence of aggressive pituitary tumors generally reported (58). However, for 94% of patients, the tumor was not aggressive, confirming that the size is not the predominant factor of aggressiveness (15, 58). Pituitary carcinomas, defined as non-contiguous craniospinal or distant metastasis, were not observed in the present series. New MRI features associated with the aggressiveness emerged from this study, including “sphenoid arch” invasion aspect and intradural extension through the cavernous sinus roof-top. Further studies are needed to confirm these preliminary results.

Limitations of this study include the monocentric evaluation, with two experimented neurosurgeons (>200 skull base surgeries per year) operating on. To which extent these results can be extrapolated remains to be determined. Of note, concentration of expertise is essential for the optimal management of such complex cases (19, 21, 57, 59–62). With 63 patients, the cohort size is limited. However, giant pituitary tumors are rare, and this is one of the largest and well characterized cohort so far. Additional studies gathering multicentric data of giant pituitary adenomas are required to extend the present results.

In conclusion, with a high efficiency on tumor volume reduction and a limited number of severe complications, pituitary surgery remains the cornerstone of giant pituitary tumors management in multidisciplinary centers of excellence. Tumor control is achieved for the majority of patients.

## Data availability statement

The raw data supporting the conclusions of this article will be made available by the authors, without undue reservation.

## Ethics statement

The studies involving human participants were reviewed and approved by Ethical Review Committee for publications of the Cochin University Hospital (CLEP, N°: AAA-2022-08022).

Written informed consent for participation was not required for this study in accordance with the national legislation and the institutional requirements.

## Author contributions

SG: conception, acquisition, analysis and interpretation of data, drafting the work, provide approval for publication. SA: acquisition and analysis of data. CV: conception of the work, interpretation of data. AJ: statistical analysis of data, revising the work critically for important intellectual content. M-LR-S: critical revision of the work. LF: critical revision of the work. PV: critical revision of the work. FB: critical revision of the work. AD: critical revision of the work. JB: critical revision of the work, provide approval for publication. GA: analysis and interpretation of data, revising the work critically for important intellectual content, provide approval for publication, agree to be accountable for all aspects of the work in ensuring that questions related to the accuracy or integrity of any part of the work are appropriately investigated and resolved. BB: conception, acquisition, analysis and interpretation of data, drafting the work, provide approval for publication. All authors contributed to the article and approved the submitted version.

## Conflict of interest

The authors declare that the research was conducted in the absence of any commercial or financial relationships that could be construed as a potential conflict of interest.

## Publisher’s note

All claims expressed in this article are solely those of the authors and do not necessarily represent those of their affiliated organizations, or those of the publisher, the editors and the reviewers. Any product that may be evaluated in this article, or claim that may be made by its manufacturer, is not guaranteed or endorsed by the publisher.

## Supplementary material

Supplementary Figure 1 Cumulative incidence of patients requiring chemotherapy after radiotherapy (Kaplan–Meyer representation).
