# Supplementary figures and images for: Outcome of giant pituitary tumors requiring surgery
Source: Front Endocrinol (Lausanne). 2022 Aug 29;13:975560. doi: 10.3389/fendo.2022.975560 (PMC9465329; doi:10.3389/fendo.2022.975560)

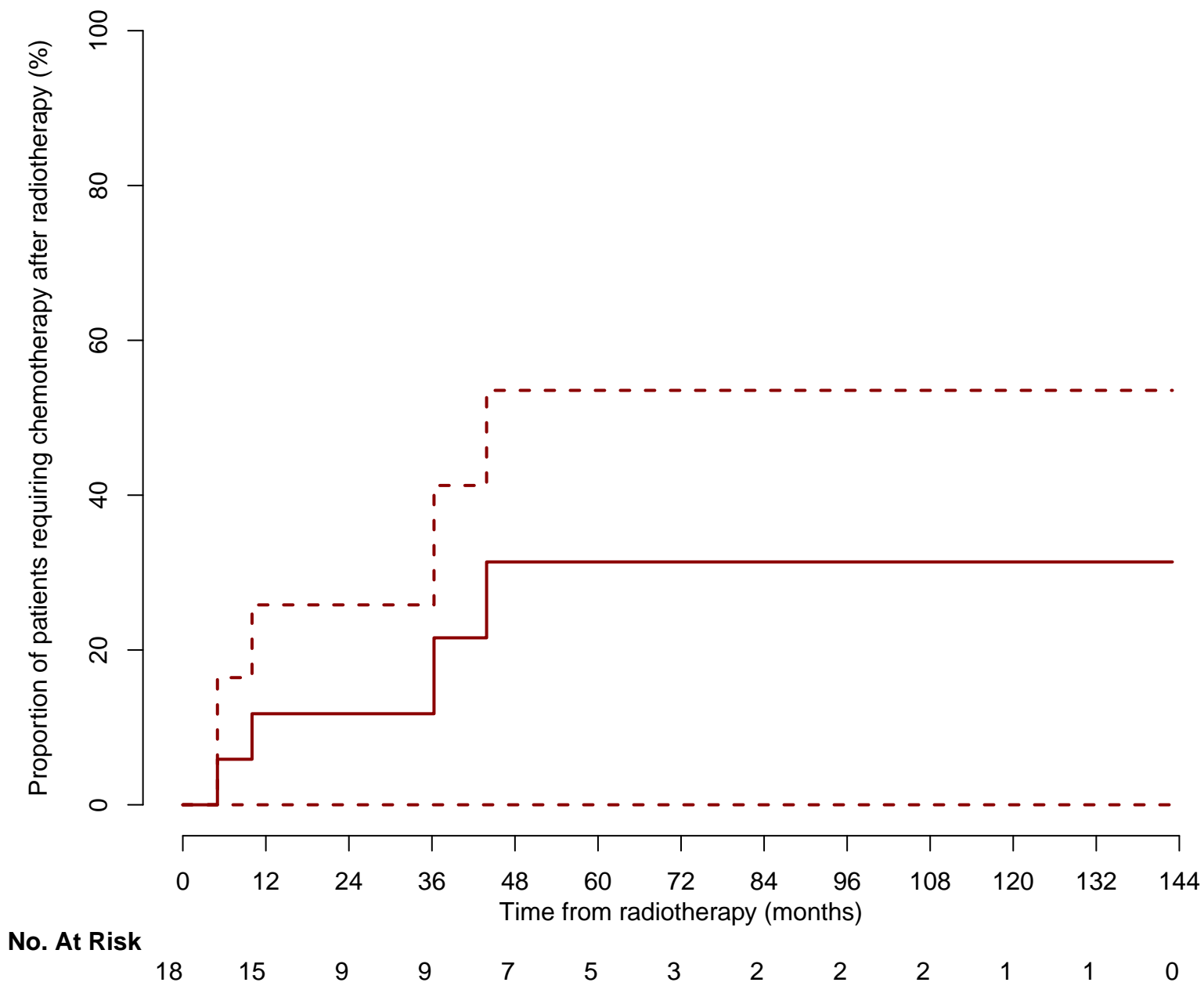

Supplement: Supplementary Figure 1 — Cumulative incidence of patients requiring chemotherapy after radiotherapy (Kaplan–Meyer representation). [file DataSheet_1.pdf]
